# Supplementary material for: Neuronal dynamics of cerebellum and medial prefrontal cortex in adaptive motor timing
Source: Nat Commun. 2025 Jan 12;16:612. doi: 10.1038/s41467-025-55884-0 (PMC11725584; doi:10.1038/s41467-025-55884-0)
Supplement: Supplementary file 2 — Reporting Summary [file 41467_2025_55884_MOESM2_ESM.pdf]

Corresponding author(s): Zhenyu Gao
Last updated by author(s): Jun 27, 2024

Reporting Summary

Nature Portfolio wishes to improve the reproducibility of the work that we publish. This form provides structure for consistency and transparency in reporting. For further information on Nature Portfolio policies, see our Editorial Policies and the Editorial Policy Checklist.

Statistics

For all statistical analyses, confirm that the following items are present in the figure legend, table legend, main text, or Methods section.

n/a	Confirmed	
☐	☒	The exact sample size (*n*) for each experimental group/condition, given as a discrete number and unit of measurement
☐	☒	A statement on whether measurements were taken from distinct samples or whether the same sample was measured repeatedly
☐	☒	The statistical test(s) used AND whether they are one- or two-sided<br>*Only common tests should be described solely by name; describe more complex techniques in the Methods section.*
☒	☐	A description of all covariates tested
☐	☒	A description of any assumptions or corrections, such as tests of normality and adjustment for multiple comparisons
☐	☒	A full description of the statistical parameters including central tendency (e.g. means) or other basic estimates (e.g. regression coefficient) AND variation (e.g. standard deviation) or associated estimates of uncertainty (e.g. confidence intervals)
☐	☒	For null hypothesis testing, the test statistic (e.g. *F*, *t*, *r*) with confidence intervals, effect sizes, degrees of freedom and *P* value noted<br>*Give P values as exact values whenever suitable.*
☒	☐	For Bayesian analysis, information on the choice of priors and Markov chain Monte Carlo settings
☒	☐	For hierarchical and complex designs, identification of the appropriate level for tests and full reporting of outcomes
☒	☐	Estimates of effect sizes (e.g. Cohen's *d*, Pearson's *r*), indicating how they were calculated

Our web collection on statistics for biologists contains articles on many of the points above.

Software and code

Policy information about availability of computer code

Data collection	Behavior data was acquired using Basler camera (acA640-750um) equipped with a high-resolution lens (C125-0618-5M-P, Basler) and digitized using National Instruments System (NI 9263 and NI 9269).In-vivo electrophysiological recordings were digitized, amplified, and acquired using Intan Evaluation System (RHD2000, Intan Technology). Fluorescence microscopy images were collected using wide-field fluorescence scanner (Axio Imager 2, ZEISS). Brightfield images were taken by NanoZoomer (Hamamatsu).
Data analysis	Electrophysiological and behavior data were analyzed using Matlab 2017 and 2019. All statistics were performed using Matlab 2017, 2019, and GraphPad Prism 8.

For manuscripts utilizing custom algorithms or software that are central to the research but not yet described in published literature, software must be made available to editors and reviewers. We strongly encourage code deposition in a community repository (e.g. GitHub). See the Nature Portfolio guidelines for submitting code & software for further information.

Data

Policy information about availability of data

All manuscripts must include a data availability statement. This statement should provide the following information, where applicable:

- Accession codes, unique identifiers, or web links for publicly available datasets
- A description of any restrictions on data availability
- For clinical datasets or third party data, please ensure that the statement adheres to our policy

All the data and codes can be accessed with the allowance of the corresponding author (email to z.gao@erasmusmc.nl).

Research involving human participants, their data, or biological material

Policy information about studies with human participants or human data. See also policy information about sex, gender (identity/presentation), and sexual orientation and race, ethnicity and racism.

Reporting on sex and gender	Both sex of mice were used in this study. We do not claim any sex differences in this study. The data from both sex of mice were analyzed together.
Reporting on race, ethnicity, or other socially relevant groupings	We do not claim any impact of socially relevant groupings on this study.
Population characteristics	This study do not include any data from human research participants.
Recruitment	The mice met the including criteria were randomly recruited and blind to the researchers. We do not think this recruitment process can have impact on our results.
Ethics oversight	Erasmus laboratory animal science center

Note that full information on the approval of the study protocol must also be provided in the manuscript.

Field-specific reporting

Please select the one below that is the best fit for your research. If you are not sure, read the appropriate sections before making your selection.

☒ Life sciences ☐ Behavioural & social sciences ☐ Ecological, evolutionary & environmental sciences

For a reference copy of the document with all sections, see nature.com/documents/nr-reporting-summary-flat.pdf

Life sciences study design

All studies must disclose on these points even when the disclosure is negative.

Sample size	The sample sizes for electrophysiological and behavioral experiments were decided similar to the sample sizes used in this fields: more than 3 animals were used in each experiment, more than 10 sessions were recorded in each behavior paradigm, dozens to hundreds of neurons were recorded to access the electrophysiological dynamics in response to correlation with behavior.
Data exclusions	No data was excluded.
Replication	All results were replicated at least in multiple animals (n >= 3) with multiple sessions (n >= 10 in total).
Randomization	Randomization is not applicable in these experiments.
Blinding	Investigators were not blinded to execute experiments or to analyze data.

Reporting for specific materials, systems and methods

We require information from authors about some types of materials, experimental systems and methods used in many studies. Here, indicate whether each material, system or method listed is relevant to your study. If you are not sure if a list item applies to your research, read the appropriate section before selecting a response.

Materials & experimental systems

n/a	Involved in the study
☒	☐ Antibodies
☒	☐ Eukaryotic cell lines
☒	☐ Palaeontology and archaeology
☐	☒ Animals and other organisms
☒	☐ Clinical data
☒	☐ Dual use research of concern
☒	☐ Plants

Methods

n/a	Involved in the study
☒	☐ ChIP-seq
☒	☐ Flow cytometry
☒	☐ MRI-based neuroimaging

Animals and other research organisms

Policy information about studies involving animals; ARRIVE guidelines recommended for reporting animal research, and Sex and Gender in Research

Laboratory animals	Wild-type C57BL/6J (000664) mice were obtained from Jackson Laboratory.
Wild animals	This study did not involve wild animals.
Reporting on sex	Both male and female animals were used in this study.
Field-collected samples	This study did not involve samples collected from the field.
Ethics oversight	All animal experiments in this study were approved by the institutional animal welfare committee of Erasmus MC in accordance with Central Authority for Scientific Procedures on Animals guidelines.

Note that full information on the approval of the study protocol must also be provided in the manuscript.

Plants

Seed stocks	No plants were used in this study.
Novel plant genotypes	No plants were used in this study.
Authentication	No plants were used in this study.
